# Epidemiological profile of patients hospitalized with Crohn’s disease due to severe acute respiratory infection during the COVID-19 pandemic: a 2-year report from Brazil

**DOI:** 10.3389/fmed.2024.1440101

**Published:** 2024-10-23

**Authors:** Laís Silva Nascimento, Fernando Augusto Lima Marson, Raquel de Cássia dos Santos

**Affiliations:** ^1^Laboratory of Natural Products, São Francisco University, Bragança Paulista, Brazil; ^2^Laboratory of Molecular Biology and Genetics, São Francisco University, Bragança Paulista, Brazil; ^3^Laboratory of Clinical and Molecular Microbiology, São Francisco University, Bragança Paulista, Brazil; ^4^LunGuardian Research Group-Epidemiology of Respiratory and Infectious Diseases, São Francisco University, Bragança Paulista, Brazil

**Keywords:** COVID-19, Crohn’s disease, epidemiology, hospitalization, SARS-CoV-2

## Abstract

**Background and aims:**

The novel coronavirus-induced severe acute respiratory syndrome (COVID-19) led to one of the most significant global pandemics of the 21st century, causing substantial challenges for healthcare systems worldwide, including those in Brazil. This study aimed to investigate the demographic and clinical profiles of hospitalized patients in Brazil who had both COVID-19 and Crohn’s disease (CD) over a 2-year period.

**Methods:**

An epidemiological analysis was conducted using data from Open-Data-SUS. The study focused on describing the demographic characteristics, clinical manifestations, comorbidities, and hospitalization details of patients afflicted with severe acute respiratory syndrome due to COVID-19 and CD, with the aim of predicting mortality risk.

**Results:**

The states of São Paulo, Paraná, and Minas Gerais accounted for 50% of the reported COVID-19 cases. The most affected racial group consisted of individuals who self-declared as mixed race. Common comorbidities included heart disease, diabetes mellitus, and obesity. The age group most affected was 25 to 60 years old, particularly among hospitalized patients with both CD and COVID-19 who ultimately succumbed to the illness. A multivariable analysis was conducted to identify the following significant risk factors for death: (a) the presence of neurological disorder (OR = 6.716; 95% CI = 1.954–23.078), (b) the need for intensive care (OR = 3.348; 95% CI = 1.770–6.335), and (c) the need for invasive mechanical ventilation (OR = 59.017; 95% CI = 19.796–175.944).

**Conclusion:**

There was no discernible gender-based prevalence among hospitalized patients with CD and COVID-19; however, individuals of mixed race were disproportionately affected. The 25 to 60 age group emerged as the most vulnerable demographic group, with high risks of hospitalization and mortality. Moreover, the study highlights the potential for COVID-19 to induce systemic pathologies that may result in long-term degenerative effects and sequelae.

## Graphical abstract

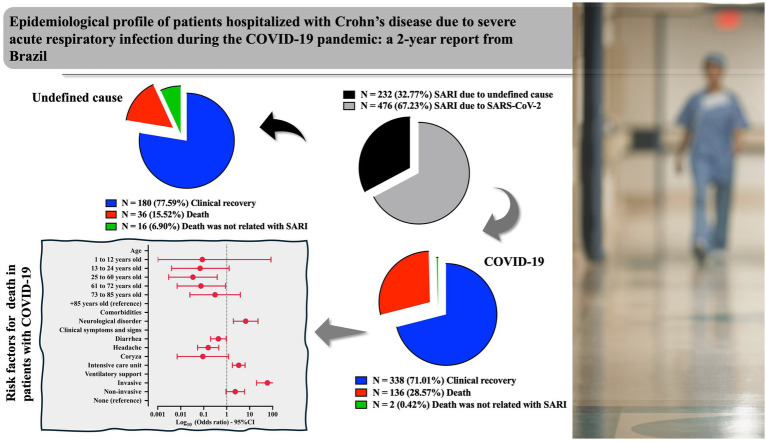

%, percentage; 95%CI, 95% confidence interval; COVID-19, coronavirus disease (COVID)-19; N, number of patients; SARI, severe acute respiratory infection; SARS-CoV-2, severe acute respiratory syndrome coronavirus 2.

## 1 Introduction

The severe acute respiratory syndrome caused by the new coronavirus (SARS-CoV-2, severe acute respiratory syndrome coronavirus 2) was responsible for one of the biggest pandemics of the 21st century, raising concerns about health care in Brazil and globally (1, 2). The SARS-CoV-2 infection can cause various clinical manifestations, such as fever, dyspnea, myalgias, and gastrointestinal problems, which may progress to death, which is associated mainly with pulmonary complications (3). The severe clinical condition is characterized by an inflammatory disorder of cytokines with hematological and coagulation changes that can lead to tissue damage and death (4). The mortality rate can be considered high and is associated with social factors, older age, and the presence of comorbidities (for example, immunosuppression, diabetes mellitus, heart disease, and systemic arterial hypertension) (5).

Since the beginning of the COVID-19 pandemic, the number of studies addressing developing and redirecting new therapies has been growing rapidly. SARS-CoV-2 causes more than just respiratory symptoms; recent studies have concluded that high levels of endogenous chemicals are produced in response to the inflammation caused by this condition, which can lead to alterations and disturbances in target tissues, surpassing protective barriers and affecting the organism systemically (6).

Patients with cardiopulmonary diseases or metabolic comorbidities, autoimmune diseases, or those undergoing any treatment that may compromise their immunity (such as chemotherapy, radiotherapy, or corticosteroid therapy) are at a higher risk of death (7). Inflammatory bowel disease affects more than five million people worldwide, and in Brazil, an increase in the number of new cases has been observed in recent years. The incidence of inflammatory bowel disease is 13.30 new cases per 100,000 inhabitants annually (8). Crohn’s disease (CD) and ulcerative colitis are two major forms of inflammatory bowel disease, both of which are idiopathic, chronic, and autoimmune in origin (9), with a tendency to relapse over time (10). These conditions are thought to be triggered by a dysregulated immune response, resulting in inflammation that damages the intestinal walls, affecting multiple layers and mucous membranes, and causing lesions (11).

In CD, a defect in the intestinal barrier is primarily observed, leading to increased permeability to antigens, which triggers an exaggerated immune response and chronic inflammation. However, the alteration of the intestinal mucosal barrier alone is insufficient for disease development. Genetic, socioenvironmental, microbiological, and immunological factors also play critical roles as risk factors in the onset and progression of the disease (12).

Inflammatory bowel diseases also exhibit extraintestinal manifestations, which are often more problematic than intestinal ones and may or may not correlate with clinical activity. Pulmonary involvement is infrequent, usually asymptomatic or related to changes in respiratory function tests, but bronchiectasis and pneumonia are more common in ulcerative colitis. It is important to note that many respiratory manifestations of inflammatory bowel diseases are related to the immunosuppressive and immunobiological drugs used (13, 14). Therapeutic interventions for CD often include immunomodulatory agents to regulate the aberrant immune response. However, these treatments inherently increase the risk of infections, potentially predisposing individuals to severe COVID-19 manifestations due to compromised immune function (15).

The COVID-19 pandemic has had widespread and multifaceted impacts on global health, which continues to be the subject of ongoing research. Restrictions on movement and contact reduced adherence to treatment, and the introduction of new vaccines have all contributed to heightened levels of stress (16–18). Psychological stress and adverse psychosocial conditions are known to exacerbate intestinal inflammatory (19), potentially increasing relapse rates in patients with inflammatory bowel diseases, even those with quiescent disease. Consequently, these factors can accelerate the progression of inflammatory bowel diseases (20, 21). Notably, stress is a well-documented factor that substantially contributes to the exacerbation of CD (22, 23). During the COVID-19 pandemic, heightened stress levels have been reported in the general population, potentially affecting the trajectory of disease activity (24, 25).

Despite the clinical significance of this intersection, studies elucidating the epidemiological profile of individuals with concurrent CD and COVID-19 are scarce. Hence, the present investigation endeavors to assess the demographic and clinical characteristics of patients hospitalized with COVID-19 and concomitant CD in Brazil over 2-years period amid the COVID-19 pandemic.

**Figure 1:**
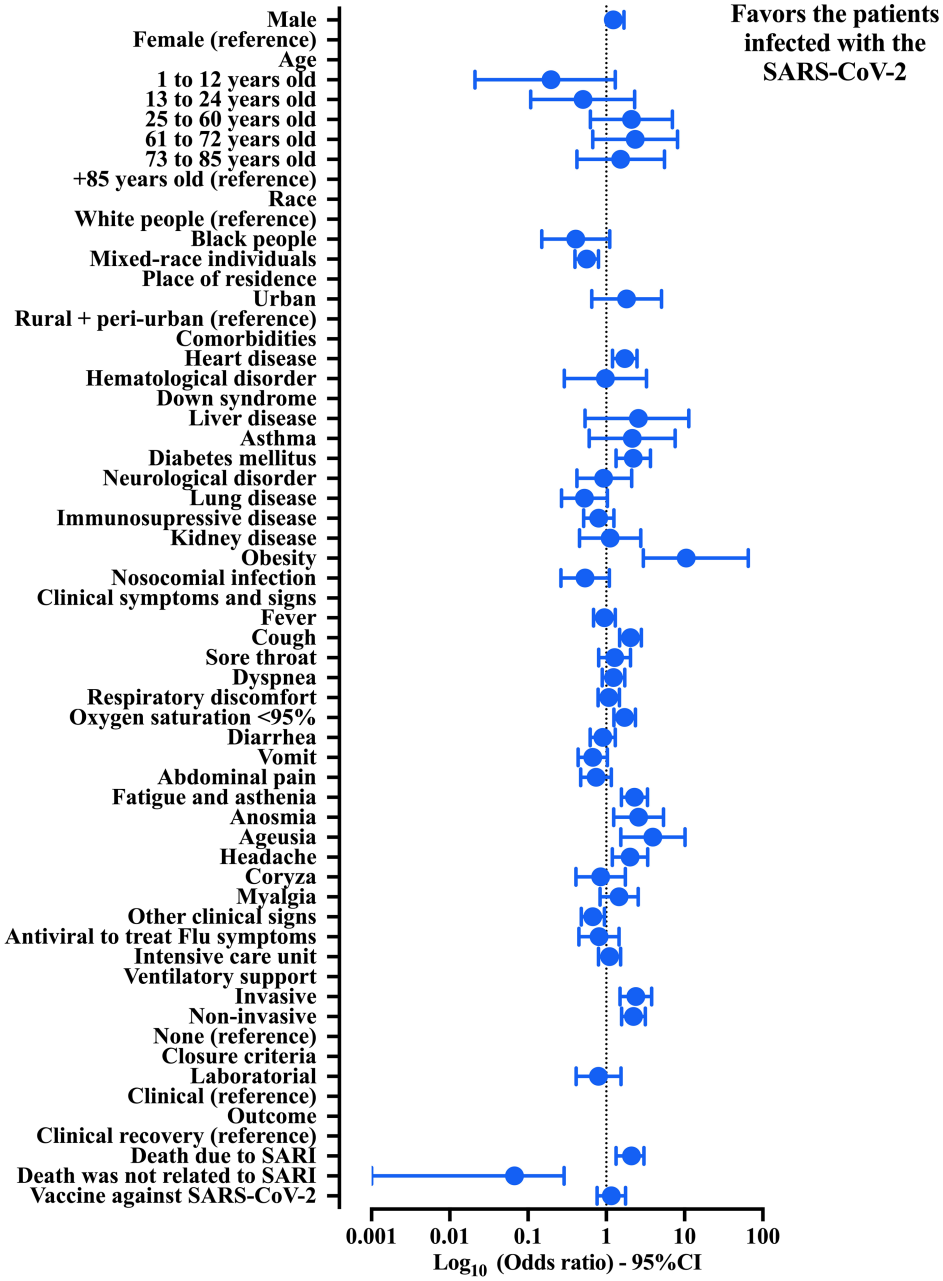
Association between the patients hospitalized with Crohn’s disease (CD) and severe acute respiratory infection (SARI) grouped by etiological agents regarding the demographic and clinical features. Crude data were obtained from the Open-Data SUS (https://opendatasus.saude.gov.br/). The data collection process was conducted from 29 December 2019 to 20 March 2022. 95% CI, 95% confidence interval; SARS-CoV-2, severe acute respiratory syndrome coronavirus disease 2. The statistical analysis was done using the chi-square test and Fisher exact test. It was not possible to calculate the odds ratio and the 95% CI for Down syndrome because none of patients was classified in the group of SARI due to an undefined cause.

**Figure 2:**
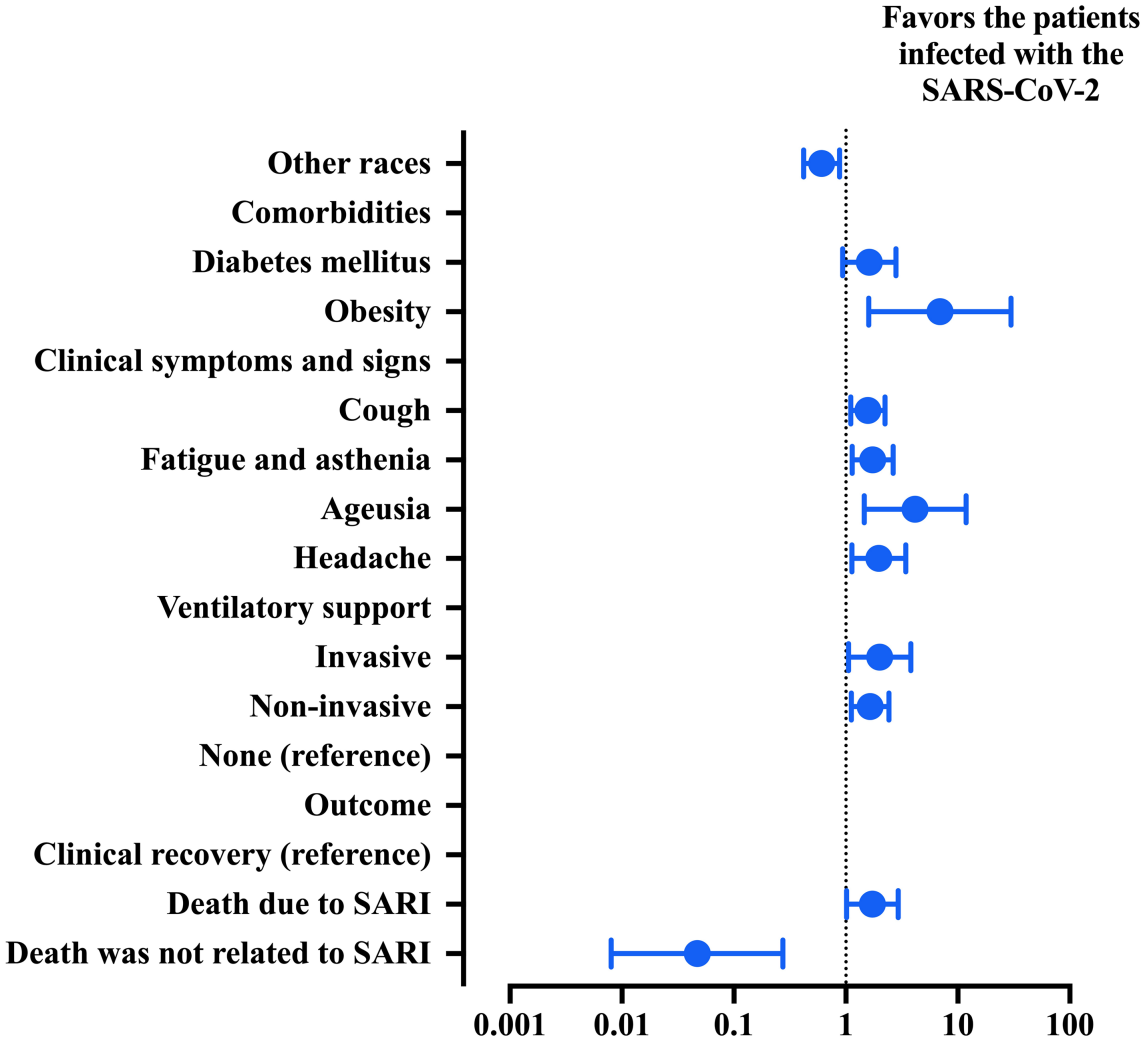
Multivariable analysis was used to describe the risk factors associated with the probability of classifying patients with Crohn’s disease (CD) into groups of patients with severe acute respiratory syndrome due to coronavirus disease (COVID)-19 or due to an undefined cause. Features included in step one: race, comorbidities (heart disease, diabetes mellitus, and obesity), clinical signs (cough, oxygen saturation below 95%, fatigue and asthenia, anosmia, and ageusia), need for ventilatory support and outcome. The statistical analysis was carried out using the binary logistic regression model with the backward stepwise method. An alpha error of 0.05 was adopted. 95% CI, 95% confidence interval; SARI, severe acute respiratory infection; SARS-CoV-2, severe acute respiratory syndrome coronavirus 2.

**Figure 3:**
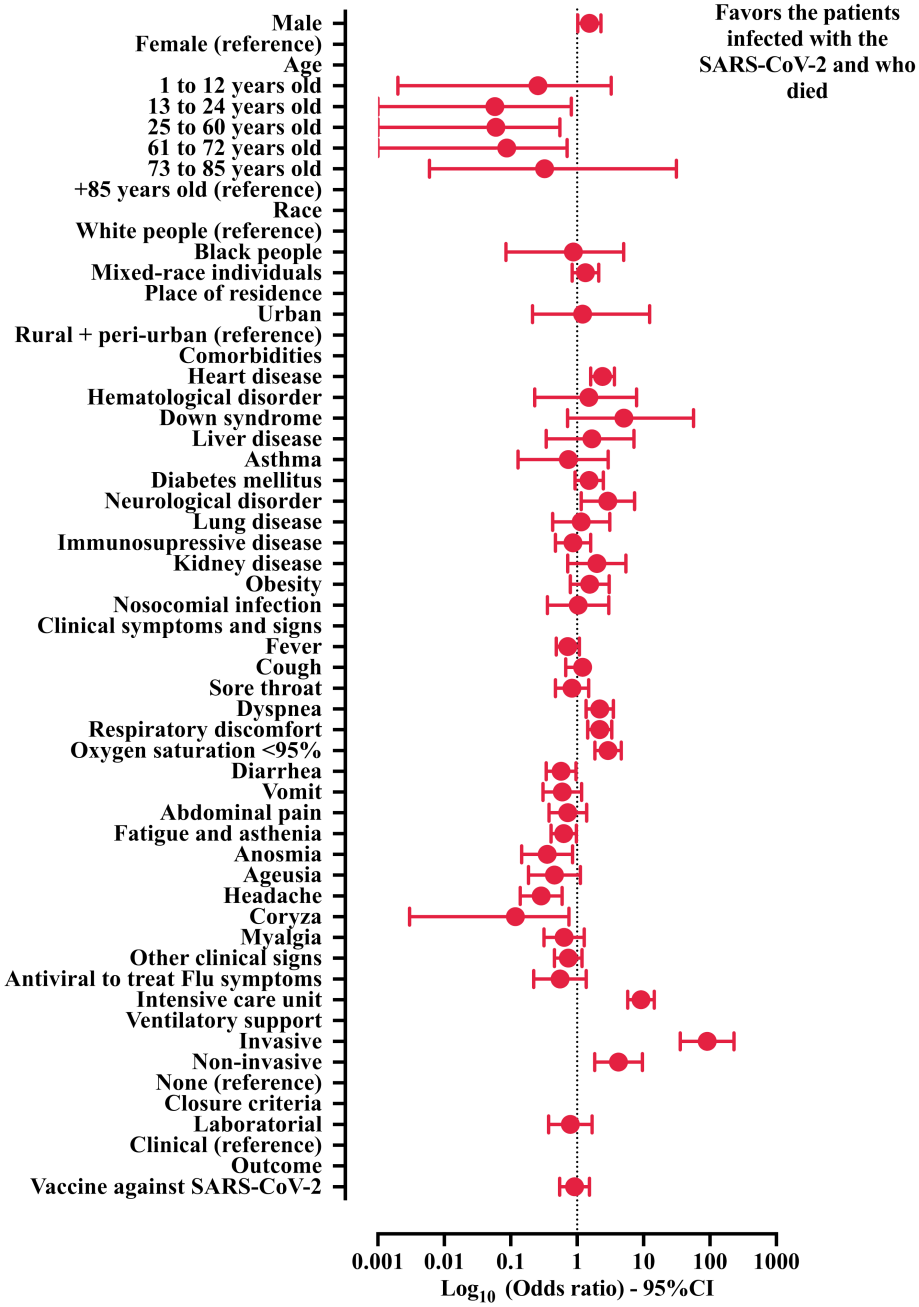
Association between demographic and clinical features and death in the patients hospitalized with Crohn’s disease (CD) and severe acute respiratory infection (SARI) due to SARS-CoV-2. Crude data was obtained from the Open-Data SUS (https://opendatasus.saude.gov.br/). The data collection process was conducted from 29 December 2019 to 20 March 2022. 95% CI, 95% confidence interval; SARS-CoV-2, severe acute respiratory syndrome coronavirus disease 2. The statistical analysis was done using the chi-square test and Fisher exact test.

**Figure 4:**
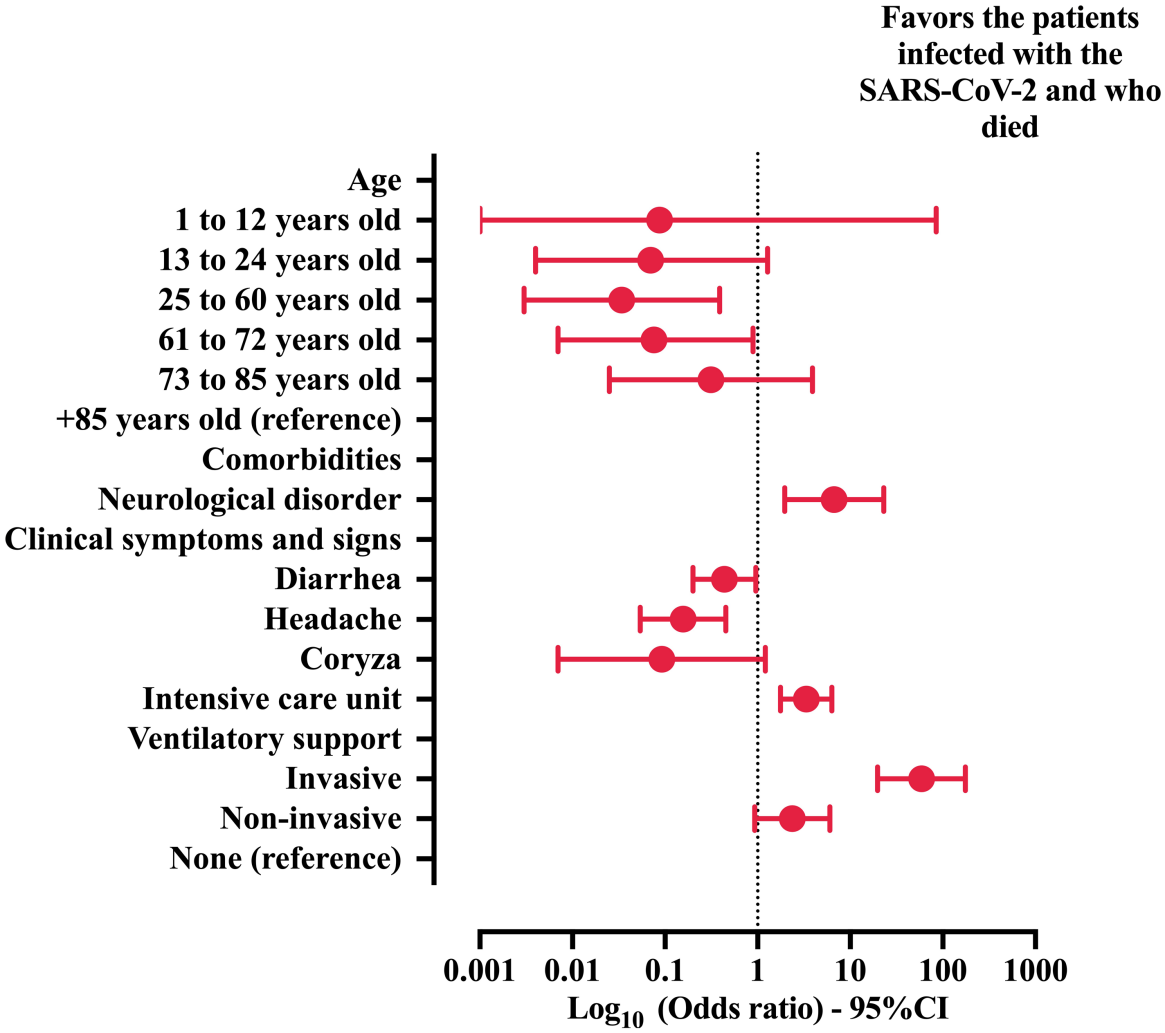
Multivariable analysis to describe the risk factors associated with the probability of death in patients with Crohn’s disease (CD) and coronavirus disease (COVID)-19. Features included in step one: sex, age, comorbidities (heart disease and neurological disorder), clinical signs (dyspnea, respiratory discomfort, oxygen saturation below 95%, diarrhea, fatigue and asthenia, anosmia, headache, coryza), the need for an intensive care unit, and the need for ventilatory support. The statistical analysis was carried out using the binary logistic regression model with the backward stepwise method. An alpha error of 0.05 was adopted. 95% CI, 95% confidence interval; SARS-CoV-2, severe acute respiratory syndrome coronavirus 2.

## 2 Methods

An epidemiological analysis was carried out using the data available at Open-Data-SUS.^1^ The Brazilian Ministry of Health compiled the data based on severe acute respiratory infection (SARI) surveillance data through the Information System Platform for Epidemiological Surveillance of Influenza (SIVEP-flu, acronym for *Sistema de Informação de Vigilância Epidemiológica da Gripe*). The SIVEP-flu dataset has been used since 2009, and it was mainly implemented due to the H1N1 virus pandemic (swine flu). It is also responsible for centralizing the SARI reports for the Brazilian Ministry of Health (26). Several previous studies also used this dataset, mainly for the COVID-19 pandemic (27–39).

The analysis was performed to evaluate two topics, namely, (i) to compare the demographic data, clinical symptoms, comorbidities, and hospitalization information according to the SARI groups [patients with COVID-19 versus hospitalized patients due to an undefined cause (possible COVID-19 underdiagnosis)]; and (ii) to identify the risk of death due to SARS-CoV-2 infection using the patients features (bivariate and multivariable analysis) regarding demographic data, clinical symptoms, comorbidities, and hospitalization information. The CD was self-reported by patients for inclusion in the database; in addition, the comorbidity was confirmed, when possible, from the information derived from clinical data or diagnoses certified by the Brazilian healthcare system because several patients were imputed from the Brazilian Unified Health System or from the institutions where the patients performed their follow-up.

The following workflow was applied in the study:

- Race classification: Although the meaning of race and ethnicity might overlap, the Merriam-Webster dictionary defines race as “a group sharing outward physical characteristics and some commonalities of culture and history”, whereas ethnicity is defined as “markers acquired from the group with which one shares cultural, traditional, and familial bonds” (38). Thus, from now on, the term race will be used and not ethnicity since it is most suitable for our variable. Moreover, the Brazilian Institute of Geography and Statistics classifies Brazilian citizens into five official races as follows: White people, Black people, individuals with multiracial backgrounds (mixed individuals), Asian individuals, and Indigenous peoples (31, 40). The race was self-declared, and the individuals should identify themselves by selecting only one category. In addition, the patients were grouped according to age using the age periods of human life, as reported by Dyussenbaye (41). In this context, the individuals were grouped as follows: (infancy period) <1 year of age, (childhood period) 1 to 12 years old, (period of youth) 13 to 24 years old, (maturity stage) 25 to 60 years old, (presenile period) 61 to 72 years old, (senile period) 73 to 85 years old, and (elderly period) +85 years old.
- Data acquisition: The authors obtained the .csv file from the Open-Data-SUS platform (see text footnote 1). The authors downloaded the file and opened it using the Statistical Package for the Social Sciences (SPSS) software (IBM SPSS Statistics for Macintosh, Version 27.0, IBM Inc., Armonk, NY, United States). Subsequently, the data collection process was conducted from 29 December 2019 to 20 March 2022. The inclusion criteria included individuals who tested positive for SARS-CoV-2 via real-time polymerase chain reaction (RT-PCR) or had an undefined agent causing SARI, and who had CD as a comorbidity. Other diseases that affected the inflammatory digestive tract were not included, as as their classification could not be obtained from the dataset.
- Dataset adjustments and data description: The dataset provided the following information: (i) demographic profile, including gender (male and female), age (<1-year-old, 1 to 12 years old, 13 to 24 years old, 25 to 60 years old, 61 to 72 years old, 73 to 85 years old, and +85 years old), and place of residence (urban, rural, and peri-urban); (ii) data for virus infection such as residing in a place with a flu outbreak, presence of hospital-acquired infection (nosocomial), and antiviral drug use to treat the influenza virus infection; (iii) presence of comorbidities [comorbidities (at least one comorbidity), heart disease, hematologic disorder, Down syndrome, hepatic disorder, asthma, diabetes mellitus, neurological disorder, systemic arterial hypertension, chronic respiratory disorders, immunosuppressive disorder, renal disease, obesity, and other comorbidities (excluding the previous ones)]; (iv) clinical symptoms related to SARI (fever, cough, sore throat, dyspnea, respiratory discomfort, oxygen saturation below 95%, diarrhea, vomit, abdominal pain, fatigue, anosmia, ageusia, myalgia, and other symptoms); (v) need for intensive care unit (ICU) treatment and need for mechanical ventilation support (invasive mechanical ventilation, non-invasive mechanical ventilation, and absence of mechanical ventilation); and (vi) outcomes (hospital discharge or death). For accuracy, the authors revised the epidemiological data from the individuals with SARI obtained in the dataset. The authors also coded the categorical data using numbers to perform the attribute of missing data and descriptive and inferential statistical analyses. The authors saved the SPSS dataset as a .xls file to perform the missing data imputation.
- Missing data analysis: The inclusion of missing data for some features was performed for the following reasons: (i) the dataset had more than 5% missing data, (ii) the dataset did not have missing data only for the dependent variable, and (iii) the authors assumed that the variables were missing completely at random. Additionally, four characteristics were excluded for having over 40.0% missingness, which included imaging exams [X-ray (75.1%) and high-resolution computed tomography (53.2%) of the chest], educational level (59.6%), residence in a place with previous flu outbreak (66.3%), and vaccination against influenza virus (60.6%). The missing data was imputed using the XLSTAT Statistical Software for Excel (Addinsoft Inc., Paris, Île-de-France, France). For the qualitative (categorical data) analysis, missing data was estimated using the NIPALS (Nonlinear Iterative Partial Least Squares) algorithm. The XLSTAT Statistical Software generated a new Excel dataset (.xls) used to perform the inferential statistical analyses in the SPSS software.
- Descriptive analysis: Descriptive analyses were carried out using the number of individuals (*N*) and the percentage (%) for categorical data. For the inferential statistical analyses, whenever possible, the odds ratio (OR) and 95% confidence interval (95% CI) were also presented (see below).
- Bivariate analysis: The authors performed the bivariate statistical analysis using the SPSS software and OpenEpi software (OpenEpi: Open-Source Epidemiologic Statistics for Public Health, Version. www.OpenEpi.com, 2013/04/06). The chi-square test and Fisher Exact test were used to evaluate the frequency of death according to the epidemiological data among the individuals with positive SARS-CoV-2 infection. The OR and 95% CI were calculated to estimate the association of each marker with the presence of death. The OR was calculated using the OpenEpi software for 2 × 2 tables, including the value for each patient’s characteristics. The authors summarized the results in tables and figures. The figures were built using GraphPad Prism version 10.2.3 for Mac (http://www.graphpad.com, GraphPad Software, San Diego, CA, United States). The same protocol was applied to compare the differences between patients grouped regarding the SARI classification, such as patients with SARS-CoV-2 or an undefined etiological agent.
- Multivariable analysis: Multivariable analysis was performed using the binary logistic regression model with the backward stepwise method. Markers with a *p*-value of ≤0.05 in the bivariate analysis were included in the regression model. The response variable was the health outcome (hospital discharge or death) or the groups for the SARI classification. The authors did not use the data for symptoms (other) and the patients’ features with a *p-*value of >0.05 in the bivariate analysis. The logistic regression model presented (i) B coefficient [including the SE (standard error)], which for the constant was called intercept; (ii) the Wald chi-square test and its significance; (iii) degrees of freedom (df) for the Wald chi-square test; and (iv) Exp(B), which represents the exponentiation of the B coefficient (OR) including its 95% CI. Before conducting the statistical analysis, the authors tested the markers for multicollinearity, using cut-offs of <0.1 for tolerance and >10 for variance inflation factor.

This study did not require approval from the ethics committee, as the data were publicly available and did not contain personal information about the individuals, thereby being exempt from ethical review.

Despite the clinical significance of this intersection, studies elucidating the epidemiological profile of individuals with concurrent CD and COVID-19 are scarce. Hence, the present investigation endeavors to assess the demographic and clinical characteristics of patients hospitalized with COVID-19 and concomitant CD in Brazil over 2-years period amid the COVID-19 pandemic.

## 3 Results

### 3.1 Epidemiological information

Table 1 shows the places of notification, residence, and hospitalization of hospitalized patients with CD and SARI in Brazil during the COVID-19 pandemic. The cases were computed in 23 states plus the Federal District; therefore, states such as Acre, Amapá, and Tocantins, all located in the northern region, were not described during the study because these states did not have any cases of CD imputed in the Open-Data-SUS. In our data, a low divergence between the state and federal districts was observed in relation to where the patients were notified, lived, and were hospitalized. Only three states accounted for over 50% of all reported cases for both SARI due to an undefined etiological agent [São Paulo (83, 35.8%), Paraná (37, 15.9%), and Minas Gerais (33, 14.2%)] and SARS-CoV-2 infection [São Paulo (174, 36.6%), Minas Gerais (63, 13.2%), and Rio Grande do Sul (48, 10.1%)]. As described before, the same profile was found for the place of residence [SARI due to an undefined etiological agent—São Paulo (82, 35.3%), Paraná (36, 15.5%), and Minas Gerais (33, 14.2%), as well as SARS-CoV-2 infection—São Paulo (171, 35.9%), Minas Gerais (64, 13.4%), and Rio Grande do Sul (48, 10.1%)] and place of hospitalization [SARI due to an undefined etiological agent—São Paulo (83, 35.8%), Paraná (37, 15.9%), and Minas Gerais (33, 14.2%), as well as SARS-CoV-2 infection—São Paulo (174, 36.6%), Minas Gerais (63, 13.2%), and Rio Grande do Sul (48, 10.1%)].

**Table 1:** Description of the places of notification, residence, and hospitalization of the patients hospitalized with Crohn’s disease (CD) and with severe acute respiratory infection (SARI) in Brazil during the coronavirus disease (COVID)-19 pandemic.

States and Federal District	Place of notification		Place of residence		Place of hospitalization	
	SARI due to an undefined cause	SARS-CoV-2	SARI due to an undefined cause	SARS-CoV-2	SARI due to an undefined cause	SARS-CoV-2
Alagoas	1 (0.4%)	4 (0.8%)	2 (0.9%)	4 (0.8%)	1 (0.4%)	4 (0.8%)
Amazonas	0 (0.0%)	3 (0.6%)	0 (0.0%)	3 (0.6%)	0 (0.0%)	3 (0.6%)
Bahia	11 (4.7%)	13 (2.7%)	11 (4.7%)	13 (2.7%)	11 (4.7%)	13 (2.7%)
Ceará	6 (2.6%)	12 (2.5%)	6 (2.6%)	12 (2.5%)	6 (2.6%)	12 (2.5%)
Federal District	1 (0.4%)	11 (2.3%)	1 (0.4%)	11 (2.3%)	1 (0.4%)	11 (2.3%)
Espírito Santo	1 (0.4%)	1 (0.2%)	1 (0.4%)	1 (0.2%)	1 (0.4%)	1 (0.2%)
Goiás	3 (1.3%)	15 (3.2%)	3 (1.3%)	15 (3.2%)	3 (1.3%)	15 (3.2%)
Maranhão	1 (0.4%)	0 (0.0%)	1 (0.4%)	1 (0.2%)	1 (0.4%)	0 (0.0%)
Minas Gerais	33 (14.2%)	63 (13.2%)	33 (14.2%)	64 (13.4%)	33 (14.2%)	63 (13.2%)
Mato Grosso do Sul	4 (1.7%)	8 (1.7%)	4 (1.7%)	8 (1.7%)	4 (1.7%)	8 (1.7%)
Mato Grosso	2 (0.9%)	4 (0.8%)	2 (0.9%)	5 (1.1%)	2 (0.9%)	4 (0.8%)
Pará	0 (0.0%)	1 (0.2%)	0 (0.0%)	1 (0.2%)	0 (0.0%)	1 (0.2%)
Paraíba	3 (1.3%)	2 (0.4%)	3 (1.3%)	2 (0.4%)	3 (1.3%)	2 (0.4%)
Pernambuco	2 (0.9%)	5 (1.1%)	2 (0.9%)	5 (1.1%)	2 (0.9%)	5 (1.1%)
Piauí	1 (0.4%)	5 (1.1%)	1 (0.4%)	4 (0.8%)	1 (0.4%)	5 (1.1%)
Paraná	37 (15.9%)	40 (8.4%)	36 (15.5%)	40 (8.4%)	37 (15.9%)	40 (8.4%)
Rio de Janeiro	11 (4.7%)	31 (6.5%)	11 (4.7%)	31 (6.5%)	11 (4.7%)	31 (6.5%)
Rio Grande do Norte	2 (0.9%)	6 (1.3%)	2 (0.9%)	6 (1.3%)	2 (0.9%)	6 (1.3%)
Rondônia	2 (0.9%)	0 (0.0%)	2 (0.9%)	0 (0.0%)	2 (0.9%)	0 (0.0%)
Roraima	1 (0.4%)	0 (0.0%)	1 (0.4%)	0 (0.0%)	1 (0.4%)	0 (0.0%)
Rio Grande do Sul	18 (7.8%)	48 (10.1%)	18 (7.8%)	48 (10.1%)	18 (7.8%)	48 (10.1%)
Santa Catarina	9 (3.9%)	28 (5.9%)	10 (4.3%)	29 (6.1%)	9 (3.9%)	28 (5.9%)
Sergipe	0 (0.0%)	2 (0.4%)	0 (0.0%)	2 (0.4%)	0 (0.0%)	2 (0.4%)
São Paulo	83 (35.8%)	174 (36.6%)	82 (35.3%)	171 (35.9%)	83 (35.8%)	174 (36.6%)

The data were described using the number (*N*) and the percentage (%) of individuals. Crude data were obtained from the Open-Data SUS (https://opendatasus.saude.gov.br/). The data collection process was conducted from 29 December 2019 to 20 March 2022. SARS-CoV-2, severe acute respiratory syndrome coronavirus 2.

### 3.2 Bivariate and multivariable analysis to determine the predictors for SARS-CoV-2 infection among those with CD and SARI

The association of hospitalized patients with CD and SARI according to the SARS-CoV-2 infection diagnosis is shown below (Table 2 and Figure 1). Our data revealed a low prevalence of female sex (50.4%) and a higher incidence of patients in adult age (25 to 60 years old) (59.6%). Sex and age were not associated with grouping according to the SARS-CoV-2 infection diagnosis. White people presented the highest prevalence (70.8%), and when the White people were compared with the mixed individuals, a low risk of SARS-CoV-2 infection was described (OR = 0.562; 95% CI = 0.397–0.796). Moreover, the majority of the patients lived in urban areas (97.9%) without association with the probability of classification to a positive SARS-CoV-2 diagnosis.

**Table 2:** Association between the patients hospitalized with Crohn’s disease (CD) and severe acute respiratory infection (SARI) grouped by etiological agents regarding the demographic and clinical features.

Marker	Data	SARI due to an undefined cause	SARS-CoV-2 (reference)	Total	*p*	Odds ratio	95% CI
Sex	Male	107 (46.1%)	244 (51.3%)	351 (49.6%)	0.199	1.229	0.971 to 1.683
	Female	125 (53.9%)	232 (48.7%)	357 (50.4%)		1	Reference
Age	1 to 12 years old	14 (6.0%)	3 (0.6%)	17 (2.4%)	0.105	0.197	0.021 to 1.304
	13 to 24 years old	33 (14.2%)	20 (4.2%)	53 (7.5%)	0.303	0.505	0.108 to 2.302
	25 to 60 years old	120 (51.7%)	302 (63.4%)	422 (59.6%)	0.219	2.097	0.628 to 7.001
	61 to 72 years old	36 (15.5%)	101 (21.2%)	137 (19.4%)	0.311	2.338	0.672 to 8.130
	73 to 85 years old	24 (10.3%)	44 (9.2%)	68 (9.6%)	0.517	1.528	0.421 to 5.532
	+85 years old	5 (2.2%)	6 (1.3%)	11 (1.6%)		1	Reference
Race	White people	145 (62.5%)	356 (74.8%)	501 (70.8%)		1	Reference
	Black people	8 (3.4%)	8 (1.7%)	16 (2.3%)	0.069	0.407	0.150 to 1.106
	Asian	0 (0.0%)	3 (0.6%)	3 (0.4%)		NA	NA
	Mixed-race individuals	79 (34.1%)	109 (22.9%)	188 (26.6%)	**0.001**	**0.562**	**0.397 to 0.796**
Place of residence	Urban	225 (97.0%)	468 (98.3%)	693 (97.9%)	0.271	1.820	0.652 to 5.081
	Rural + peri-urban	7 (3.0%)	8 (1.7%)	15 (2.1%)		1	Reference
Comorbidities							
Heart disease	Yes	53 (22.8%)	161 (33.8%)	214 (30.2%)	**0.003**	**1.726**	**1.204 to 2.476**
	No	179 (77.2%)	315 (66.2%)	494 (69.8%)		1	Reference
Hematological disorder	Yes	4 (1.7%)	8 (1.7%)	12 (1.7%)	1.000	0.974	0.290 to 3.269
	No	228 (98.3%)	468 (98.3%)	696 (98.3%)		1	Reference
Down syndrome	Yes	0 (0.0%)	6 (1.3%)	6 (0.8%)	0.185	NA	NA
	No	232 (100.0%)	470 (98.7%)	702 (99.2%)		NA	NA
Liver disease	Yes	2 (0.9%)	10 (2.1%)	12 (1.7%)	0.355	2.468	0.536 to 11.350
	No	230 (99.1%)	466 (97.9%)	696 (98.3%)		1	Reference
Asthma	Yes	3 (1.3%)	13 (2.7%)	16 (2.3%)	0.289	2.143	0.605 to 7.596
	No	229 (98.7%)	463 (97.3%)	692 (97.7%)		1	Reference
Diabetes mellitus	Yes	21 (9.1%)	86 (18.1%)	107 (15.1%)	**0.002**	**2.216**	**1.336 to 3.673**
	No	211 (90.9%)	390 (81.9%)	601 (84.9%)		1	Reference
Neurological disorder	Yes	10 (4.3%)	19 (4.0%)	29 (4.1%)	0.842	0.923	0.422 to 2.108
	No	222 (95.7%)	457 (96.0%)	679 (95.9%)		1	Reference
Lung disease	Yes	17 (7.3%)	19 (4.0%)	36 (5.1%)	0.068	0.526	0.268 to 1.032
	No	215 (92.7%)	457 (96.0%)	672 (94.9%)		1	Reference
Immunosuppressive disease	Yes	36 (15.5%)	61 (12.8%)	97 (13.7%)	0.352	0.800	0.513 to 1.249
	No	196 (84.5%)	415 (87.2%)	611 (86.3%)		1	Reference
Kidney disease	Yes	7 (3.0%)	16 (3.4%)	23 (3.2%)	0.808	1.118	0.454 to 2.756
	No	225 (97.0%)	460 (96.6%)	685 (96.8%)		1	Reference
Obesity	Yes	2 (0.9%)	40 (8.4%)	42 (5.9%)	**<0.001**	**10.530**	**2.979 to 65.300**
	No	230 (99.1%)	436 (91.6%)	666 (94.1%)		1	Reference
Nosocomial infection	Yes	15 (6.5%)	17 (3.6%)	32 (4.5%)	0.082	0.536	0.263 to 1.093
	No	217 (93.5%)	459 (96.4%)	676 (95.5%)		1	Reference
Clinical symptoms and signs							
Fever	Yes	140 (60.3%)	281 (59.0%)	421 (59.5%)	0.739	0.947	0.688 to 1.304
	No	92 (39.7%)	195 (41.0%)	287 (40.5%)		1	Reference
Cough	Yes	114 (49.1%)	316 (66.4%)	430 (60.7%)	**<0.001**	**2.044**	**1.484 to 2.815**
	No	118 (50.9%)	160 (33.6%)	278 (39.3%)		1	Reference
Sore throat	Yes	28 (12.1%)	71 (14.9%)	99 (14.0%)	0.305	1.277	0.799 to 2.041
	No	204 (87.9%)	405 (85.1%)	609 (86.0%)		1	Reference
Dyspnea	Yes	149 (64.2%)	328 (68.9%)	477 (67.4%)	0.212	1.235	0.886 to 1.719
	No	83 (35.8%)	148 (31.1%)	231 (32.6%)		1	Reference
Respiratory discomfort	Yes	121 (52.2%)	257 (54.0%)	378 (53.4%)	0.646	1.077	0.786 to 1.474
	No	111 (47.8%)	219 (46.0%)	330 (46.6%)		1	Reference
Oxygen saturation	<95%	111 (47.8%)	291 (61.1%)	402 (56.8%)	**<0.001**	**1.715**	**1.249 to 2.354**
	≥95%	121 (52.2%)	185 (38.9%)	306 (43.2%)		1	Reference
Diarrhea	Yes	57 (24.6%)	108 (22.7%)	165 (23.3%)	0.579	0.901	0.624 to 1.302
	No	175 (75.4%)	368 (77.3%)	543 (76.7%)		1	Reference
Vomit	Yes	41 (17.7%)	60 (12.6%)	101 (14.3%)	0.070	0.672	0.436 to 1.035
	No	191 (82.3%)	416 (87.4%)	607 (85.7%)		1	Reference
Abdominal pain	Yes	36 (15.5%)	57 (12.0%)	93 (13.1%)	0.190	0.741	0.472 to 1.162
	No	196 (84.5%)	419 (88.8%)	615 (86.9%)		1	Reference
Fatigue and asthenia	Yes	42 (18.1%)	160 (33.6%)	202 (28.5%)	**<0.001**	**2.291**	**1.559 to 3.364**
	No	190 (81.9%)	316 (66.4%)	506 (71.5%)		1	Reference
Anosmia	Yes	9 (3.9%)	45 (9.5%)	54 (7.6%)	**0.009**	**2.587**	**1.242 to 5.388**
	No	223 (96.1%)	431 (90.5%)	654 (92.4%)		**1**	**Reference**
Ageusia	Yes	5 (2.2%)	38 (8.0%)	43 (6.1%)	**0.002**	**3.939**	**1.529 to 10.140**
	No	227 (97.8%)	438 (92.0%)	665 (93.9%)		**1**	**Reference**
Headache	Yes	20 (8.6%)	76 (16.0%)	96 (13.6%)	**0.007**	**2.014**	**1.197 to 3.387**
	No	212 (91.4%)	400 (84.0%)	612 (86.4%)		1	Reference
Coryza	Yes	12 (5.2%)	21 (4.4%)	33 (4.7%)	0.652	0.846	0.409 to 1.751
	No	220 (94.8%)	455 (95.6%)	675 (95.3%)		1	Reference
Myalgia	Yes	18 (7.8%)	52 (10.9%)	70 (9.9%)	0.185	1.458	0.832 to 2.554
	No	214 (92.2%)	424 (89.1%)	638 (90.1%)		1	Reference
Other clinical signs	Yes	78 (33.6%)	121 (25.4%)	199 (28.1%)	**0.023**	**0.673**	**0.478 to 0.947**
	No	154 (66.4%)	355 (74.6%)	509 (71.9%)		1	Reference
Antiviral to treat flu symptoms	Yes	19 (8.2%)	32 (6.7%)	51 (7.2%)	0.479	0.808	0.448 to 1.459
	No	213 (91.8%)	444 (93.3%)	657 (92.8%)		1	Reference
Intensive care unit	Yes	84 (36.2%)	183 (38.4%)	267 (37.7%)	0.564	1.100	0.795 to 1.523
	No	148 (63.8%)	293 (61.6%)	441 (62.3%)		1	Reference
Ventilatory support	Invasive	34 (14.7%)	99 (20.8%)	133 (18.8%)	**<0.001**	**2.382**	**1.496 to 3.794**
	Non-invasive	90 (38.8%)	245 (51.5%)	335 (47.3%)	**<0.001**	**2.227**	**1.568 to 3.161**
	None	108 (46.6%)	132 (27.7%)	240 (33.9%)		1	Reference
Closing criteria	Laboratorial	219 (94.4%)	443 (93.1%)	662 (93.5%)	0.501	0.797	0.411 to 1.545
	Clinical	13 (5.6%)	33 (6.8%)	46 (6.5%)		1	Reference
Outcome	Clinical recovery	180 (77.6%)	338 (71.0%)	518 (73.2%)		1	Reference
	Death due to SARI	36 (15.5%)	136 (28.6%)	172 (24.3%)	**<0.001**	**2.012**	**1.336 to 3.030**
	Death was not related to SARI	16 (6.9%)	2 (0.4%)	18 (2.5%)	**<0.001**	**0.067**	**0.001 to 0.289**
Vaccine against SARS-CoV-2	Yes	38 (16.4%)	88 (18.5%)	126 (17.8%)	0.491	1.158	0.763 to 1.758
	No	194 (83.6%)	388 (81.5%)	582 (82.2%)		1	Reference

The data were described using the number (*N*) and the percentage (%) of individuals. Crude data were obtained from the Open-Data SUS (https://opendatasus.saude.gov.br/). Data collection comprised the period from 29 December 2019 to 20 March 2022. 95% CI, 95% confidence interval; NA, not applicable; *p*, *p*-value; SARS-CoV-2, severe acute respiratory syndrome coronavirus disease 2. The statistical analysis was conducted using the chi-square test and Fisher exact test. An alpha error of 0.05 was adopted. The significant *p*-values were presented using the bold type.

The most prevalent comorbidities were heart disease (30.2%), diabetes mellitus (15.1%), immunosuppressive disease (13.7%), obesity (5.9%), and lung disease (5.1%). However, only three were associated with a higher probability of being classified as a positive case for SARS-CoV-2 infection as follows: (a) heart disease (OR = 1.726, 95% CI = 1.204–2.476), (b) diabetes mellitus (OR = 2.216, 95% CI = 1.226–3.673), and (c) obesity (OR = 10.530, 95% CI = 2.979–65.300) (Table 2 and Figure 1).

The nosocomial infection occurred only in 32 (4.5%) of the cases. The main clinical symptoms and signs were respiratory and involved the presence of dyspnea (67.4%), cough (60.7%), fever (59.5%), oxygen saturation below 95% (56.8%), and respiratory discomfort (53.4%). Among them, only two were associated with the SARS-CoV-2 infection, namely cough (OR = 2.044; 95% CI = 1.484–2.815) and oxygen saturation below 95% (OR = 1.715; 95% CI = 1.249–2.354). In addition, other clinical symptoms and signs with a low prevalence were more common among those classified as patients with SARS-CoV-2, which included fatigue and asthenia (28.5%—OR = 2.291; 95% CI = 1.559–3.364), headache (13.6%—OR = 2.014; 95% CI = 1.197–3.387), anosmia (7.6%—OR = 2.587; 95% CI = 1.242–5.388), and ageusia (6.1%—OR = 3.939; 95% CI = 1.529–10.140). Finally, the presence of other clinical symptoms and signs (28.1%) was more frequent in patients without a confirmatory test for SARS-CoV-2 infection (OR = 0.673; 95% CI = 0.478–0.947) (Table 2 and Figure 1).

The use of antiviral drugs to treat flu symptoms and the need for ICUs occurred respectively in 51 (7.2%) and 267 (37.7%) cases without association with the SARS-CoV-2 diagnosis. Most of the patients needed invasive (18.8%) or non-invasive (47.3%) ventilatory support. Both mechanical ventilatory modes were more frequent in patients infected with the SARS-CoV-2 [(invasive ventilatory support) OR = 2.382; 95% CI = 1.496–3.794; (non-invasive ventilatory support) OR = 2.227; 95% CI = 1.568–3.161]. In our study population, 172 (24.3%) patients died due to SARI progression, and 18 (2.5%) died, but this outcome was unrelated to SARI. Death due to SARI was more common in patients with COVID-19 diagnosis (OR = 2.012; 95% CI = 1.336–3.30), while death unrelated to SARI occurred predominantly in patients with SARI due to other causes rather than SARS-CoV-2 infection (OR = 0.067; 95% CI = 0.001–0.289). Only 126 (17.8%) of the patients were vaccinated against COVID-19, and the vaccination was not associated with the group’s classification (Table 2 and Figure 1).

A multivariable analysis was conducted (Table 3 and Figure 2) to describe the risk factors associated with the likelihood of classifying patients with CD into the groups of patients with SARI. In brief, the predictors of SARS-CoV-2 infection were (a) obesity (OR = 6.922; 95% CI = 1.605–29.849), (b) cough (OR = 1.574; 95% CI = 1.107–2.238), (c) fatigue and asthenia (OR = 1.731; 95% CI = 1.137–2.637), (d) ageusia (OR = 4.156; 95% CI = 1.459–11.839), (e) headache (OR = 1.973; 95% CI = 1.133–3.436), (f) need for ventilatory support [invasive (OR = 2.005; 95% CI = 1.059–3.796) and non-invasive (OR = 1.647; 95% CI = 1.117–2.431) ventilatory support], and (g) death due to SARI (OR = 1.724; 95% CI = 1.014–2.931). However, two factors were protective against the COVID-19 diagnosis, namely, (a) race (OR = 0.606; 95% CI = 0.419–0.878; other than whites’ people) and (b) death unrelated to SARI (OR = 0.047; 95% CI = 0.008–0.273).

**Table 3:** Multivariable analysis used to describe the risk factors associated with the probability of classifying patients with Crohn’s disease (CD) into groups of patients with severe acute respiratory syndrome due to the coronavirus disease (COVID)-19 or due to an undefined cause.

Markers^a^	*B*	SE	Wald	df	*p*	Odds ratio	95% CI	
							Lower	Upper
Race (other than whites’ people)	−0.500	0.189	7.017	1	**0.008**	**0.606**	**0.419**	**0.878**
Diabetes mellitus	0.482	0.279	2.993	1	0.084	1.619	0.938	2.796
Obesity	1.935	0.746	6.733	1	**0.009**	**6.922**	**1.605**	**29.849**
Cough	0.454	0.180	6.384	1	**0.012**	**1.574**	**1.107**	**2.238**
Fatigue and asthenia	0.549	0.215	6.533	1	**0.011**	**1.731**	**1.137**	**2.637**
Ageusia	1.424	0.534	7.111	1	**0.008**	**4.156**	**1.459**	**11.839**
Headache	0.680	0.283	5.764	1	**0.016**	**1.973**	**1.133**	**3.436**
Ventilatory support								
Invasive	0.696	0.326	4.561	1	**0.033**	**2.005**	**1.059**	**3.796**
Non-invasive	0.499	0.198	6.329	1	**0.012**	**1.647**	**1.117**	**2.431**
None			7.965	2	**0.019**			
Outcome								
Death due to SARI	0.545	0.271	4.048	1	**0.044**	**1.724**	**1.014**	**2.931**
Death was not related to SARI	−3.063	0.899	11.593	1	**<0.001**	**0.047**	**0.008**	**0.273**
Clinical recovery			18.092	2	**<0.001**			
Constant	2.124	0.538	15.585	1	**<0.001**	**8.365**		

a The features included in step one: race, comorbidities (heart disease, diabetes mellitus, and obesity), clinical signs (cough, oxygen saturation below 95%, fatigue and asthenia, anosmia, and ageusia), need for ventilatory support and outcome.

The statistical analysis was carried out using the binary logistic regression model with the backward stepwise method. An alpha error of 0.05 was adopted. The significant p-values were presented using the bold type. 95% CI, 95% confidence interval; B, beta coefficient; df, degrees of freedom; p, p-values; SARI, severe acute respiratory infection; SE, standard error.

### 3.3 Bivariate and multivariable analyses to determine the predictors of death among patients infected with SARS-CoV-2

The association of hospitalized patients with CD infected with SARS-CoV-2 according to the outcomes is demonstrated (Table 4 and Figure 3). In our data, the male sex was identified as an independent marker for a higher probability of death (58.8% versus 41.2%—OR = 1.534; 95% CI = 1.025–2.294). Furthermore, using patients aged +85 years (3.7% versus 0.3%), the following age categories were considered as protective groups—13 to 24 years old (2.9% versus 4.7%—OR = 0.058; 95% CI = 0.001–0.710), 25 to 60 years old (50.7% versus 68.6%—OR = 0.060; 95% CI = 0.001–0.550), and 61 to 72 years old (22.1% versus 21.0%—OR = 0.087; 95% CI = 0.001–0.820). In addition, neither race nor place of residence was associated with the probability of death (Table 4 and Figure 3).

**Table 4:** Association between demographic and clinical features and death in the patients hospitalized with Crohn’s disease (CD) and severe acute respiratory infection (SARI) due to severe acute respiratory syndrome coronavirus 2 (SARS-CoV-2).

Marker	Data	Hospital discharge	Death due to SARI^a^	Total	*p*	Odds ratio	95% CI
Sex	Male	163 (48.2%)	80 (58.8%)	243 (51.3%)	**0.037**	**1.534**	**1.025 to 2.294**
	Female	175 (51.8%)	56 (41.2%)	231 (48.7%)		1	Reference
Age	1 to 12 years old	1 (0.3%)	1 (0.7%)	2 (0.4%)	0.929	0.258	0.002 to 31.28
	13 to 24 years old	16 (4.7%)	4 (2.9%)	20 (4.2%)	**0.019**	**0.058**	**0.001 to 0.710**
	25 to 60 years old	232 (68.6%)	69 (50.7%)	301 (63.5%)	**0.007**	**0.060**	**0.001 to 0.550**
	61 to 72 years old	71 (21.0%)	30 (22.1%)	101 (21.3%)	**0.028**	**0.087**	**0.001 to 0.820**
	73 to 85 years old	17 (5.0%)	27 (19.9%)	44 (9.3%)	0.570	0.324	0.006 to 3.267
	+85 years old	1 (0.3%)	5 (3.7%)	6 (1.3%)		1	Reference
Race	White people	258 (76.3%)	98 (72.1%)	356 (75.1%)		1	Reference
	Black people	6 (1.8%)	2 (1.5%)	8 (1.7%)	1.000	0.878	0.085 to 5.016
	Asian	3 (0.9%)	0 (0.0%)	3 (0.6%)		NA	NA
	Mixed-race individuals	71 (21.0%)	36 (26.5%)	107 (22.6%)	0.222	1.335	0.840 to 2.122
Place of residence	Urban	332 (98.2%)	134 (98.5%)	466 (98.3%)	1	1.210	0.213 to 12.410
	Rural + peri-urban	6 (1.8%)	2 (1.5%)	8 (1.7%)		1	Reference
Comorbidities							
Heart disease	Yes	95 (28.1%)	66 (48.5%)	161 (34.0%)	**<0.001**	**2.412**	**1.598 to 3.639**
	No	243 (71.9%)	70 (51.5%)	313 (66.0%)		1	Reference
Hematological disorder	Yes	5 (1.5%)	3 (2.2%)	8 (1.7%)	0.695	1.501	0.230 to 7.838
	No	333 (98.5%)	133 (97.8%)	466 (98.3%)		1	Reference
Down syndrome	Yes	2 (0.6%)	4 (2.9%)	6 (1.3%)	0.059	5.071	0.717 to 56.680
	No	336 (99.4%)	132 (97.1%)	468 (98.7%)		1	Reference
Liver disorder	Yes	6 (1.8%)	4 (2.9%)	10 (2.1%)	0.483	1.675	0.342 to 7.192
	No	332 (98.2%)	132 (97.1%)	464 (97.9%)		1	Reference
Asthma	Yes	10 (3.0%)	3 (2.2%)	13 (2.7%)	0.766	0.740	0.129 to 2.935
	No	328 (97.0%)	133 (97.8%)	461 (97.3%)		1	Reference
Diabetes mellitus	Yes	55 (16.3%)	31 (22.8%)	86 (18.1%)	0.096	1.519	0.927 to 2.489
	No	283 (83.7%)	105 (77.2%)	388 (81.9%)		1	Reference
Neurological disorder	Yes	9 (2.7%)	10 (7.4%)	19 (4.0%)	**0.019**	**2.901**	**1.152 to 7.306**
	No	329 (97.3%)	126 (92.6%)	455 (96.0%)		1	Reference
Lung disease	Yes	13 (3.8%)	6 (4.4%)	19 (4.0%)	0.776	1.154	0.429 to 3.100
	No	325 (96.2%)	130 (95.6%)	455 (96.0%)		1	Reference
Immunosuppressive disorder	Yes	45 (13.3%)	16 (11.8%)	61 (12.9%)	0.649	0.868	0.472 to 1.596
	No	293 (86.7%)	120 (88.2%)	413 (87.1%)		1	Reference
Kidney disorder	Yes	9 (2.7%)	7 (5.1%)	16 (3.4%)	0.176	1.984	0.724 to 5.438
	No	329 (97.3%)	129 (94.9%)	458 (96.6%)		1	Reference
Obesity	Yes	25 (7.4%)	15 (11.0%)	40 (8.4%)	0.198	1.552	0.791 to 3.044
	No	313 (92.6%)	121 (89.0%)	434 (91.6%)		1	Reference
Nosocomial infection	Yes	12 (3.6%)	5 (3.7%)	17 (3.6%)	0.947	1.037	0.358 to 3.001
	No	326 (96.4%)	131 (96.3%)	457 (96.4%)		1	Reference
Clinical symptoms and signs							
Fever	Yes	208 (61.5%)	73 (53.7%)	281 (59.3%)	0.115	0.724	0.485 to 1.083
	No	130 (38.5%)	63 (46.3%)	193 (40.7%)		1	Reference
Cough	Yes	224 (66.3%)	91 (66.9%)	315 (66.5%)	0.894	1.029	0.675 to 1.570
	No	114 (33.7%)	45 (33.1%)	159 (33.5%)		1	Reference
Sore throat	Yes	52 (15.4%)	18 (13.2%)	70 (14.8%)	0.551	0.839	0.471 to 1.494
	No	286 (84.6%)	118 (86.8%)	404 (85.2%)		1	Reference
Dyspnea	Yes	219 (64.8%)	109 (80.1%)	328 (69.2%)	**0.001**	**2.194**	**1.362 to 3.534**
	No	119 (35.2%)	27 (19.9%)	146 (30.8%)		1	Reference
Respiratory discomfort	Yes	165 (48.8%)	92 (67.6%)	257 (54.2%)	**<0.001**	**2.192**	**1.444 to 3.329**
	No	173 (51.2%)	44 (32.4%)	217 (45.8%)		1	Reference
Oxygen saturation	<95%	185 (54.7%)	106 (77.9%)	291 (61.4%)	**<0.001**	**2.922**	**1.848 to 4.622**
	≥95%	153 (45.3%)	30 (22.1%)	183 (38.6%)		1	Reference
Diarrhea	Yes	85 (25.1%)	22 (16.2%)	107 (22.6%)	**0.035**	**0.574**	**0.342 to 0.965**
	No	253 (74.9%)	114 (83.8%)	367 (77.4%)		1	Reference
Vomit	Yes	47 (13.9%)	12 (8.8%)	59 (12.4%)	0.166	0.599	0.307 to 1.168
	No	291 (86.1%)	124 (91.2%)	415 (87.6%)		1	Reference
Abdominal pain	Yes	43 (12.7%)	13 (9.6%)	56 (11.8%)	0.335	0.725	0.377 to 1.396
	No	295 (87.3%)	123 (90.4%)	418 (88.2%)		1	Reference
Fatigue and asthenia	Yes	123 (36.4%)	36 (26.5%)	159 (33.5%)	**0.039**	**0.629**	**0.405 to 0.978**
	No	215 (63.6%)	100 (73.5%)	315 (66.5%)		1	Reference
Anosmia	Yes	39 (11.5%)	6 (4.4%)	45 (9.5%)	**0.017**	**0.354**	**0.146 to 0.856**
	No	299 (88.5%)	130 (95.6%)	429 (90.5%)		1	Reference
Ageusia	Yes	31 (9.2%)	6 (4.4%)	37 (7.8%)	0.081	0.457	0.186 to 1.122
	No	307 (90.8%)	130 (95.6%)	437 (92.2%)		1	Reference
Headache	Yes	67 (19.8%)	9 (6.6%)	76 (16.0%)	**<0.001**	**0.287**	**0.139 to 0.593**
	No	271 (80.2%)	127 (93.4%)	398 (84.0%)		1	Reference
Coryza	Yes	20 (5.9%)	1 (0.7%)	21 (4.4%)	**0.012**	**0.118**	**0.003 to 0.753**
	No	318 (94.1%)	135 (99.3%)	453 (95.6%)		1	Reference
Myalgia	Yes	41 (12.1%)	11 (8.1%)	52 (11.0%)	0.203	0.638	0.317 to 1.281
	No	297 (87.9%)	125 (91.9%)	422 (89.0%)		1	Reference
Other clinical signs	Yes	91 (26.9%)	29 (21.3%)	120 (25.3%)	0.205	0.736	0.457 to 1.184
	No	247 (73.1%)	107 (78.7%)	354 (74.7%)		1	Reference
Antiviral to treat flu signs	Yes	26 (7.7%)	6 (4.4%)	32 (6.8%)	0.198	0.554	0.223 to 1.377
	No	312 (92.3%)	130 (95.6%)	442 (93.2%)		1	Reference
Intensive care unit	Yes	81 (24.0%)	101 (74.3%)	182 (38.4%)	**<0.001**	**9.156**	**5.789 to 14.480**
	No	257 (76.0%)	35 (25.7%)	292 (61.6%)		1	Reference
Ventilatory support	Invasive	16 (4.7%)	82 (60.3%)	98 (20.7%)	**<0.001**	**90.79**	**35.79 to 230.3**
	Non-invasive	198 (58.6%)	47 (34.6%)	245 (51.7%)	**<0.001**	**4.205**	**1.843 to 9.596**
	None	124 (36.7%)	7 (5.1%)	131 (27.6%)		1	Reference
Closing criteria	Laboratorial	316 (93.5%)	125 (91.9%)	441 (93.0%)	0.552	0.791	0.373 to 1.679
	Clinical	22 (6.5%)	11 (8.1%)	33 (7.0%)		1	Reference
Vaccine against SARS-CoV-2 infection	Yes	64 (18.9%)	24 (17.6%)	88 (18.6%)	0.744	0.917	0.547 to 1.540
	No	274 (81.1%)	112 (82.4%)	386 (81.4%)		1	Reference

a Two patients that died were excluded due to other causes different from the SARS-CoV-2 infection.

The data were described using the number (*N*) and the percentage (%) of individuals. Crude data were obtained from the Open-Data SUS (https://opendatasus.saude.gov.br/). The data collection process was conducted from 29 December 2019 to 20 March 2022. 95% CI, 95% confidence interval; *p*, *p*-value. NA, not applicable. The statistical analysis was done using the chi-square test and Fisher exact test. An alpha error of 0.05 was adopted. The significant *p*-values were presented using the bold type.

Among comorbidities, only two were associated with a higher probability of death: neurological disorder (OR = 2.901; 95% CI = 1.152–7.306) and heart disease (OR = 2.412; 95% CI = 1.598–3.639). In addition, among the clinical symptoms and signs, three were associated with a higher probability of death [(oxygen saturation below 95%) OR = 2.922; 95% CI = 1.848–4.622, (dyspnea) OR = 2.194; 95% CI = 1.362–3.544, and (respiratory discomfort) OR = 2.192; 95% CI = 1.444–3.329], while five symptoms were associated with a lower probability of death [(coryza) OR = 0.118; 95% CI = 0.003–0.753, (headache) OR = 0.287; 95% CI = 0.139–0.593, (anosmia) OR = 0.354; 95% CI = 0.146–0.856, (diarrhea) OR = 0.574; 95% CI = 0.342–0.965], and (fatigue and asthenia) OR = 0.629; 95% CI = 0.405–0.978. The presence of nosocomial infection and the use of antiviral drugs to treat the flu symptoms were not associated with the probability of death (Table 4 and Figure 3).

The need for ICU treatment (OR = 9.156; 95% CI = 5.789–14.480) and invasive (OR = 90.79; 95% CI = 35.79–230.3) or non-invasive (OR = 4.205; 95% CI = 1.843–9.596) mechanical ventilation were associated with a higher probability of death. Curiously, the vaccines against COVID-19 did not influence the outcomes (Table 4 and Figure 3).

A multivariable analysis was conducted to describe the risk factors associated with the likelihood of classifying patients with CD into the groups with a higher probability of death (Table 5 and Figure 4). In brief, the predictors of death were (a) the presence of neurological disorder (OR = 6.716; 95% CI = 1.954–23.078), (b) the need for ICU (OR = 3.348; 95% CI = 1.770–6.335), and (c) the need for invasive mechanical ventilatory support (OR = 59.017; 95% CI = 19.796–175.944). In contrast, the following markers were protective against death: (a) age [(25 to 60 years old) OR = 0.034; 95% CI = 0.003–0.389, and (61 to 72 years old) OR = 0.076; 95% CI = 0.007–0.890], (b) diarrhea (OR = 0.437; 95% CI = 0.200–0.958), and (c) headache (OR = 0.157; 95% CI = 0.054–0.453).

**Table 5:** Multivariable analysis to describe the risk factors associated with the probability of death in hospitalized patients with Crohn’s disease (CD) and severe acute respiratory infection (SARI) due to severe acute respiratory syndrome coronavirus 2 (SARS-CoV-2).

Markers^a^	*B*	SE	Wald	df	*p*	Odds ratio	95% CI	
							Lower	Upper
Age								
1 to 12 years old	−2.438	3.510	0.482	1	0.487	0.087	<0.001	84.851
13 to 24 years old	−2.653	1.480	3.216	1	0.073	0.070	0.004	1.280
25 to 60 years old	−3.375	1.241	7.398	1	**0.007**	**0.034**	**0.003**	**0.389**
61 to 72 years old	−2.576	1.255	4.214	1	**0.040**	**0.076**	**0.007**	**0.890**
73 to 85 years old	−1.165	1.292	0.813	1	0.367	0.312	0.025	3.923
+85 years old (reference)			27.135	5	**<0.001**			
Neurological disorder	1.904	0.630	9.145	1	**0.002**	**6.716**	**1.954**	**23.078**
Diarrhea	−0.827	0.400	4.273	1	**0.039**	**0.437**	**0.200**	**0.958**
Headache	−1.854	0.542	11.694	1	**<0.001**	**0.157**	**0.054**	**0.453**
Coryza	−2.390	1.321	3.273	1	0.070	0.092	0.007	1.221
Intensive care unit	1.208	0.325	13.800	1	**<0.001**	**3.348**	**1.770**	**6.335**
Ventilatory support								
Invasive	4.078	0.557	53.535	1	**<0.001**	**59.017**	**19.796**	**175.944**
Non-invasive	0.860	0.476	3.263	1	0.071	2.363	0.929	6.009
None (reference)			75.919	2	**<0.001**			
Constant	0.117	1.250	0.009	1	0.925	1.124		

a Features included in step one: sex, age, comorbidities (heart disease and neurological disorder), clinical signs (dyspnea, respiratory discomfort, oxygen saturation below 95%, diarrhea, fatigue and asthenia, anosmia, headache, coryza), the need for the intensive care unit and the need for ventilatory support.

The statistical analysis was carried out using the binary logistic regression model with the backward stepwise method. An alpha error of 0.05 was adopted. The significant *p*-values were presented using the bold type. 95% CI, 95% confidence interval: *B*, beta coefficient; df, degrees of freedom; *p*, *p*-values; SE, standard error.

### 3.4 Association between the probability of death in hospitalized patients with CD and control individuals from other studies previously published

The study conducted a comparative analysis of case fatality rates between patients with CD and control cohorts from literature, all using the Open-Data-SUS database. The comparison focused on hospitalized patients with severe acute respiratory syndrome due to SARS-CoV-2 infection (Table 6). In 2020, patients with CD had a mortality rate of 28.7% (136/475), while control patients had a mortality rate of 33.9% (214,027/630,000), with a statistically significant *p*-value of 0.017 and an OR of 0.786 (95% CI = 0.644–0.959), indicating a lower risk of death for patients with CD. Similarly, in 2021, patients with CD maintained the same mortality rate (28.7%), whereas the control group had a mortality rate of 33.3% (390,192/1,170,000), with a *p*-value of 0.023 and an OR of 0.795 (95% CI = 0.651–0.970), again reflecting a reduced mortality risk for patients with CD. In 2022, the mortality rate for patients with CD remained at 28.7%, while the control group had a mortality rate of 29.3% (65,711/224,000). The *p*-value of 0.757 indicated no significant difference (OR = 0.969, 95% CI = 0.794–1.183). However, in 2023, patients with CD still had a mortality rate of 28.7%, while the control group had a reduced mortality rate of 24.4% (3,444/14,100), with a significant *p*-value of 0.032 and an OR of 1.248 (95% CI = 1.109–1.528), suggesting a higher mortality risk for patients with CD in that year. Over the entire period, patients with CD exhibited a cumulative mortality rate of 28.7%, compared to 33.2% in controls (673,374/2,025,000), with a *p*-value of 0.039 and an OR of 0.811 (95% CI = 0.665–0.989), suggesting an overall lower mortality risk for patients with CD across the pandemic, particularly in 2020 and 2021. The study underscores the temporal variability in mortality risk for patients with CD and emphasizes the importance of ongoing surveillance for this vulnerable population.

**Table 6:** Association between the probability of death in hospitalized patients with Crohn’s disease (CD) and control individuals from other studies previously published.

Comparative study	Groups	Patients with CD [*N* (%)]	Other studies [*N* (%)]	*p*-value	OR	95% CI
Sansone et al., 2022^a^	Death	136 (28.7%)	574,887 (34.8%)	**<0.001**	**0.754**	**0.618–0.921**
	Hospital discharge	338 (71.3%)	1,077,870 (65.2%)			
Sansone et al., 2022^b^	Death	136 (28.7%)	73,723 (26.3%)	0.234	1.129	0.925–1.378
	Hospital discharge	338 (71.3%)	206,810 (73.7%)			
Boscheiro et al., 2022^c^	Death	136 (28.7%)	25,571 (14.0%)	**<0.001**	**2.468**	**2.022–3.13**
	Hospital discharge	338 (71.3%)	156,871 (86.0%)			
Palamim et al., 2023^d^—2020	Death	136 (28.7%)	214,027 (33.9%)	**0.017**	**0.786**	**0.644–0.959**
	Hospital discharge	338 (71.3%)	417,893 (66.1%)			
Palamim et al., 2023^d^—2021	Death	136 (28.7%)	390,192 (33.3%)	**0.023**	**0.795**	**0.651–0.970**
	Hospital discharge	338 (71.3%)	770,806 (66.7%)			
Palamim et al., 2023^d^—2022	Death	136 (28.7%)	65,711 (29.3%)	0.757	0.969	0.794–1.183
	Hospital discharge	338 (71.3%)	158,253 (70.7%)			
Palamim et al., 2023^d^—2023	Death	136 (28.7%)	3,444 (24.4%)	**0.032**	**1.248**	**1.109–1.528**
	Hospital discharge	338 (71.3%)	10,682 (75.6%)			
Palamim et al., 2023^d^—Total	Death	136 (28.7%)	673,374 (33.2%)	**0.039**	**0.811**	**0.665–0.989**
	Hospital discharge	338 (71.3%)	1,357,634 (66.8%)			

All the patients were hospitalized in Brazil due to the presence of severe acute respiratory syndrome caused by the coronavirus disease (COVID)-19. The data were described using the number (N) and percentage (%) of individuals. The significant p-values were presented using the bold type.

a The study comprised only the first year of the COVID-19 pandemic when the highest number of deaths (case fatality rates) were observed in Brazil due to the inefficiency of the Brazilian government in dealing with the impact of the COVID-19 pandemic, mainly in the Brazilian Health System (62).

b The study also comprised only the first year of the COVID-19 pandemic when the highest number of deaths (case fatality rates) were observed in Brazil due to the inefficiency of the Brazilian government in dealing with the impact of the COVID-19 pandemic, mainly in the Brazilian Health System (61). However, in this study, only patients without comorbidities were enrolled.

c The study also comprised only the first year of the COVID-19 pandemic, when the highest number of deaths (case fatality rates) were observed in Brazil due to the inefficiency of the Brazilian government in dealing with the impact of the COVID-19 pandemic, mainly in the Brazilian Health System (30). However, in this study, only patients without comorbidities were enrolled, and the control sample was restrictive in terms of severity.

d The study comprised an overview of the COVID-19 pandemic over 4 years, and the data were presented by the year of notification (39). %, percentage; 95% CI, 95% confidence interval; *N*, number of individuals; OR, odds ratio. The statistical analysis was performed using the chi-square test.

## 4 Discussion

The onset of the COVID-19 pandemic in March 2020 triggered a global surge in transmission and contamination, culminating in a significant toll of fatalities and overwhelming pressure on healthcare infrastructure. This resulted in a high number of deaths and an intense strain on the healthcare system. Hospitals were reorganized due to the increased need for beds, especially for patients requiring intensive care (1).

Since the onset of the COVID-19 pandemic, numerous studies have focused on accelerated diagnostic methods and the exploration and repurposing of therapeutic approaches. However, the potential long-term effects on individuals who have contracted the virus remain uncertain. COVID-19 extends beyond being a respiratory illness. As demonstrated by Jin et al. (42), the virus-induced inflammatory response triggers the production of endogenous chemicals that can breach tissue barriers and spread systemically through hematogenic transmission pathways (43).

Giustino et al. (44) reported increased levels of pro-inflammatory cytokines—interleukin 6 (IL-6), interleukin 1 (IL-1), and tumor necrosis factor-alpha (TNF-*α*)—in COVID-19-positive patients. These cytokines can trigger immune cell activation in response to vascular changes, leading to increased blood adhesion and coagulation. Consequently, this signaling cascade activates immune cells involved in chronic inflammatory processes, potentially causing pulmonary degeneration, pulmonary fibrosis, loss of function, hypoxemia, and anoxia, which can contribute to unfavorable clinical outcomes.

Patients with cardiopulmonary or metabolic comorbidities, autoimmune diseases such as CD described in our study, or those undergoing treatments that may compromise their immunity (for example, chemotherapy, radiotherapy, or corticosteroid therapy) are at a higher risk of death (45).

The severity of cases and potential consequences in COVID-19 hospitalized patients are important markers for assessing risks and possible prognosis, especially for patients with pre-existing conditions who were left without adequate care due to the suspension of outpatient treatments (46). The pathophysiology of COVID-19 is complex, affecting various organs and systems. The lungs, considered the target organs of the respiratory system, undergo gradual functional failure, as evidenced by hypoxia and pathological findings from minimal and non-invasive autopsies on patients (47).

The pandemic significantly and disproportionately affected the Brazilian population, marked by geographical differences, socioeconomic inequalities, and low availability of therapeutic resources and healthcare teams. These factors can be important determinants in a pandemic, hindering access to available resources and leading to higher-case fatalities. Furthermore, the unequal distribution of COVID-19 cases among Brazilian regions might have resulted from underreporting (39). The state of São Paulo reported the highest number of notified cases, being a developed state with the main financial and corporate center in South America, which makes it the most influential Brazilian city on the global stage (48).

Although SARS-CoV-2 does not exhibit contagious selectivity, the impacts of infection can manifest differently depending on race, class, and gender. In this study, we observed that individuals of mixed race were the most affected. The Brazilian population, which is the fifth-largest in the world estimated at 211,293 million inhabitants, is classified as White people (45.22%), mixed race (45.06%), Black people (8.86%), Asians (0.47%), and Indigenous peoples (0.38%), which may explain the higher incidence in mixed-race patients (49).

This study focused on analyzing hospitalizations of patients with CD, a chronic and recurrent metabolic disorder that may predispose the body to other comorbidities, including a potentially positive relationship with colorectal cancer, the third leading cause of death. Patients with CD and COVID-19 have been discussed (50). CD, a chronic remittent inflammatory bowel disease, is influenced by genetic predisposition and lifestyle factors and exacerbated by urbanization and industrialization, which disrupt intestinal homeostasis and provoke immune responses, leading to chronic inflammation. This significantly impacts patients’ quality of life (51, 52). The CD also has extraintestinal manifestations, including musculoskeletal, ocular, and vascular complications such as cardiovascular disease, thromboembolism, and portal vein thrombosis, contributing to morbidity and mortality (53). Immunosuppressants are commonly used to reduce gastrointestinal inflammation but can increase susceptibility to opportunistic infections and severe illnesses due to compromised immune function (54). A substantial proportion of COVID-19 cases progress to acute respiratory distress syndrome due to critical lung injury. Hyperinflammation following SARS-CoV-2 infection is a key mechanism leading to respiratory failure, multi-organ dysfunction, and mortality (55). The pandemic has raised concerns about managing inflammatory bowel disease patients, as they may experience more severe COVID-19 due to a dysregulated immune state and immunosuppressive medication use (56, 57).

Early epidemiological data indicate a worse prognosis and higher mortality for patients with COVID-19 and chronic diseases, such as heart disease, a relevant complication to our study group. According to the literature, the extreme inflammatory response caused by COVID-19 can lead to cardiac injury through multiple disorders, causing endothelial injury and myocarditis. The cardiovascular system is affected in various ways by severe respiratory tract infections caused by SARS-CoV-2. According to the study by Giustino et al. (44), myocardial injury occurred in 25% of hospitalized COVID-19 patients and is associated with an increased risk of mortality. Increasing clinical and epidemiological evidence has shown that cardiovascular comorbidities are widely associated with an increased risk of death caused by COVID-19.

In our study, another prevalent comorbidity was neurological disorders. According to Mao et al. (56), the short-and long-term impacts of SARS-CoV-2 infection on the central nervous system remain unclear. However, the most commonly reported symptoms of COVID-19 include ageusia, anosmia, hearing loss, headaches, spasms, seizures, confusion, visual impairment, dizziness, decreased consciousness, nausea and vomiting, hemiplegia, ataxia, stroke, and cerebral hemorrhage.

The pathophysiology of SARS-CoV-2 infection involves various essential organ systems for maintaining homeostasis. The direct effect of SARS-CoV-2 hyperinflammation induces the production of endogenous substances that alter vascular hemostasis. Blood coagulation is directly affected by the release of pro-coagulant and pro-inflammatory cytokines, activating disseminated intravascular coagulation and the formation of thromboembolic states that can aggressively affect various tissues, especially those more sensitive to ischemic processes, such as lung, cardiovascular, and cerebrovascular tissues.

According to Zhong et al. (12), inflammatory complications involving the digestive system are not uncommon in individuals affected by COVID-19. Clinical manifestations such as diarrhea, nausea, vomiting, abdominal pain, gastrointestinal bleeding, lack of appetite, and constipation were reported in patients affected by the novel coronavirus, supporting our study, especially in the group that had both COVID-19 and CD, with diarrhea being the main symptom.

Studies suggest that gastrointestinal symptoms caused by SARS-CoV-2 occur because the infection is associated with the lung-intestine-brain axis, where the virus activates intestinal receptors, inducing tissue inflammation and causing a high viral load that leads to the previously mentioned gastrointestinal problems (58).

The infection induces alterations and diminishes the colonies of intestinal microorganisms, precipitating the activation of immune cells and instigating the secretion of pro-inflammatory cytokines. Consequently, this disruption in the individual’s microbiota composition, termed dysbiosis, fosters an inflammatory milieu that amplifies systemic inflammation (59, 60). Based on current studies (30, 39, 61, 62), no clear consensus has been reached regarding the impact of COVID-19 on mortality risk in patients with CD. Further research is required to comprehensively assess this relationship.

Patients with autoimmune diseases, when affected by SARS-CoV-2 infection, are at a higher risk of developing severe forms of the disease (63). Immunosuppression can lead to increased production of interferons and interleukins, activation of granulocytes, and tumor necrosis factor, resulting in intravascular inflammation, which causes alterations in angiogenesis and coagulation. In addition to understanding the immune response to COVID-19, the association of symptoms with autoimmune diseases suggests that SARS-CoV-2 may trigger secondary diseases associated with a temporary state of immunosuppression and the presence of the virus.

### 4.1 Limitations

The present study has several limitations. The key limitations include the following: (a) reliance on a national database, which may contain reporting bias, (b) potential inaccuracies in the diagnosis of CD, (c) limited external validity, as the data are derived exclusively from patients in Brazil, (d) exclusively information regarding the number of COVID-19 vaccinations received, and thus, their potential effect on clinical outcomes in patients with SARI, (e) the absence of crucial epidemiological and clinical markers (e.g., smoking status and daily living habits) in the Open-Data-SUS, which could influence clinical outcomes, (f) the unclear clinical state of the patients with CD at the time of data entry, making it impossible to determine whether the patient was in an exacerbation or remission phase of the disease, and (g) a small sample size, which may increase the risk of a Type II error.

## 5 Conclusion

The findings of this study revealed that only three Brazilian states, São Paulo, Paraná, and Minas Gerais, accounted for half of the reported cases of COVID-19. Notably, among patients concurrently hospitalized with CD and COVID-19, there was no discernible sex predilection; however, individuals self-identified as mixed-race were the most affected demographic group. The predominant comorbidities observed in these patients included heart disease, diabetes mellitus, and obesity. Additionally, clinical manifestations included symptoms such as cough, oxygen saturation below 95%, fatigue, asthenia, anosmia, ageusia, and headache. An age-based comparison indicated that individuals aged 25 to 60 years were disproportionately affected by both diseases, with this age group exhibiting increased vulnerability to mortality. In addition, the study suggests that the systemic effects of COVID-19 may lead to degenerative changes and prolonged consequences, highlighting the need for continued research into the long-term implications of the disease.

## Data Availability

The datasets presented in this study can be found in online repositories. The names of the repository/repositories and accession number(s) can be found at: https://opendatasus.saude.gov.br/.

## Footnotes

^1^ https://opendatasus.saude.gov.br/
